# Soluble ST2 and risk of cognitive impairment after acute ischemic stroke: a prospective observational study

**DOI:** 10.1186/s12877-021-02288-6

**Published:** 2021-05-24

**Authors:** Yinwei Zhu, Chongquan Fang, Qi Zhang, Yaling Lu, Rui Zhang, Aili Wang, Xiaoqing Bu, Jintao Zhang, Zhong Ju, Yonghong Zhang, Tan Xu, Chongke Zhong

**Affiliations:** 1 Department of Epidemiology, School of Public Health and Jiangsu Key Laboratory of Preventive and Translational Medicine for Geriatric Diseases, Medical College of Soochow University, 199 Renai Road, Industrial Park District, Suzhou, 215123 Jiangsu Province China; 2 Department of Epidemiology, School of Public Health, Chongqing Medical University, Chongqing, China; 3 Department of Neurology, The 88th Hospital of PLA, Taian, Shandong China; 4 Department of Neurology, Kerqin District First People’s Hospital of Tongliao City, Tongliao, Inner Mongolia China

**Keywords:** Ischemic stroke, sST2, Cognitive impairment, Montreal cognitive assessment, Mini-mental state examination

## Abstract

**Background:**

Soluble suppression of tumorigenesis-2 (sST2) was reported to be associated with cognitive performance and risk of incident stroke. However, the impact of sST2 on cognitive function after ischemic stroke is unclear. We aimed to assess the association of sST2 and cognitive impairment at 3 months in acute ischemic stroke patients.

**Methods:**

Baseline plasma sST2 levels were measured in 619 ischemic stroke patients (mean age: 60.0 ± 10.5 years) from 7 participating hospitals of the China Antihypertensive Trial in Acute Ischemic Stroke. Montreal Cognitive Assessment (MoCA) and Mini-Mental State Examination (MMSE) were used to assess cognitive status. Cognitive impairment was defined as a MoCA score < 23 or MMSE score < 27. The association between sST2 and cognitive impairment was evaluated by logistic regression analysis.

**Results:**

325 (52.5%) or 323 (52.2%) participants developed cognitive impairment according to MoCA or MMSE. After adjustment for age, sex, education, and other covariates, the odds ratio for the highest vs lowest quartile of sST2 was 2.38 (95% CI, 1.42–4.00) and 1.82 (95% CI 1.09–3.03) risk of cognitive impairment defined by MoCA and MMSE score, respectively. Incorporation sST2 into a model containing conventional risk factors significantly improved reclassification.

**Conclusions:**

Elevated plasma sST2 levels were significantly associated with post-stroke cognitive impairment.

**Supplementary Information:**

The online version contains supplementary material available at 10.1186/s12877-021-02288-6.

## Background

Stroke is a major cause of serious disability and death [1], as well as the common cause of acquired cognitive impairment [2]. Post-stroke cognitive impairment (PSCI) has been reported to be associated with unfavorable prognosis, including major disability, mortality, stroke recurrence and poorer quality of life [3–5]. Thus, novel and reliable predictors are clearly needed for early identification of patients at higher risk of PSCI.

Heart-brain axis has a greater role in the procession of cognitive impairment and dementia [6–8], available population-based evidence reported suboptimal cardiac function or abnormally elevated cardiac biomarkers, such as N-terminal pro-B-type natriuretic peptide (NT-proBNP) or high-sensitivity cardiac Troponin T (hs-cTnT), were associated with worse cognitive performance [9–13], suggesting cardiac biomarkers may be used to identify individuals at higher risk of cognitive impairment. However, whether the predictive roles of cardiac biomarkers persist in the setting of cerebrovascular disease was less consistent [7]. Soluble suppression of tumorigenesis-2 (sST2), another cardiac stress biomarker of promoting cardiomyocyte hypertrophy and fibrosis, is considered to be an important biomarker of heart failure. Recently, the Framingham Offspring showed that higher sST2 levels were associated with increased risk of incident stroke and subclinical vascular brain injury [14]. However, the association of sST2 and cognitive impairment in patients with ischemic stroke remains to be addressed.

Therefore, we aimed to prospectively assess the relationship between plasma sST2 levels in the acute phase of ischemic stroke and PSCI at 3 months using the data derived from the China Antihypertensive Trial in Acute Ischemic Stroke (CATIS).

## Materials and methods

### Study design and population

The CATIS trial, a single-blind, blinded end-points randomized clinical trial, was designed to evaluate whether immediate blood pressure (BP) reduction would reduce death and major disability at 14 days or hospital discharge. The study design and main results had been described previously [15]. The enrollment criteria were as follows: (1) first-ever ischemic stroke; (2) age ≥ 22 years; (3) time from onset to admission within 48 h; (4) systolic BP between 140 and 220 mmHg. The exclusion criteria were as follows: (1) BP ≥220/120 mmHg; (2) treated with intravenous thrombolytic therapy; (3) severe heart failure, acute myocardial infarction or unstable angina, atrial fibrillation, aortic dissection, cerebrovascular stenosis, resistant hypertension, or in a deep coma. Finally, 4071 patients with first-ever ischemic stroke within 48 h of onset and elevated systolic BP were recruited. The present prospective observational study was a pre-planned ancillary study of CATIS trial, which was designed to test cognitive function at 3 months. In the ancillary study, acute ischemic stroke patients from 7 randomly selected participating hospitals were consecutively recruited for neurological evaluations. Each of the 7 participating hospitals planned to recruit 80–100 patients. During the period of August 2009 to November 2012, 660 participants were enrolled. After further excluded participants without available cognitive evaluations (*n* = 22) or sST2 data (*n* = 19), 619 participants were finally included in the present analysis (Supplementary Figure 1).

This study protocol was approved by the ethical committee at Soochow University in China and Tulane University in the United States, as well as ethical committees at the 7 participating hospitals, in compliance with the Declaration of Helsinki. All participants provided written informed consent.

### Data collection

Fasting blood samples were drawn within 24 h of patients’ hospital admission, and were frozen − 80 °C until testing. The concentrations of plasma sST2 and serum high-sensitive C-reactive protein (hsCRP) were measured by commercially available immunoassays (R&D Systems, Minneapolis, MN). The intra-assay and inter-assay coefficients of variation for sST2 were below 5.5 and 2.4%. Similarly, the intra-assay and inter-assay coefficients of variation for hsCRP were below 8.4 and 1.6%. Laboratory technicians who performed measurements were blinded to clinical features and outcomes of patients.

Baseline data regarding demographic characteristics, clinical features, medical history, and prior use of medications were collected using a standard questionnaire at the time of enrollment. The National Institutes of Health Stroke Scale (NIHSS) and the modified Rankin Scale (mRS) were applied to assess stroke severity by trained neurologists. Trial of Org 10,172 in Acute Stroke Treatment criteria was used to classify the ischemic stroke subtypes as large-artery atherosclerosis(thrombotic), cardiac embolism (embolic) and small-vessel occlusion (lacunar), according to the symptoms and imaging data of the patients by experienced neurologists [16]. BP measurements were performed by trained nurses according to a standard protocol adapted from procedures recommended by the American Heart Association.

### Outcome assessment

Cognitive function at 3 months after stroke was evaluated by trained neurologists using the Montreal Cognitive Assessment (MoCA) and Mini-Mental State Examination (MMSE). Prior studies found that people whose education ≤12 years tended to perform worse on the MoCA test [17]. Thus, 1 point was additionally added for participants with education ≤12 years on their crude MoCA score (if < 30) to correct for education effects. Lower scores indicate worse cognitive function, and PSCI was defined as a MoCA score of less than 23 [18, 19] or MMSE score of less than 27 [20].

### Statistical analysis

Baseline characteristics were summarized according to the quartiles of plasma sST2 levels. Means with standard deviation(SD) or median with interquartile range were used for continuous variables, and the generalized linear regression models were further used to test for trend across the sST2 quartiles. Frequency with percentage was used for categorical variables, and the Cochran-Armitage trend χ^2^ tests were used to test for trend.

First, we performed generalized linear regression models to test the association between plasma sST2 levels and continuous MoCA and MMSE score. Second, both MoCA and MMSE score were categorized into two groups (without versus with PSCI). Logistic regression models were used to calculate odds ratios (ORs) and 95% confidence intervals (CIs) for the association of plasma sST2 levels with the incident of PSCI. According to baseline characteristics, previous studies and professional knowledge, three models were used with an increasing level of adjustment. Model 1 was adjusted for age, sex and education level (illiteracy, primary, high school and college or higher). Model 2 further adjusted for current smoking, alcohol drinking, time from onset to randomization, systolic BP, baseline NIHSS and mRS score, medical history (hypertension, diabetes mellitus, hyperlipidemia, and coronary heart disease), use of antihypertensive medications, randomized treatment, ischemic stroke subtype (thrombotic, embolic and lacunar), anticoagulant treatment and hypoglycemic treatment. Available evidence suggested that elevated sST2 levels were involved in pro-inflammatory response [21, 22], and inflammation may be an intermediary in the relation of sST2 levels to cognitive impairment. In consideration of adjustment for a risk factor that is in the causal pathway from the exposure to outcome will reduce or even remove the effect of interest, we further conducted a separate analysis with hsCRP included in a multivariable-adjusted model based on the model 2. We tested for linear trends across the median of sST2 quartiles.

Moreover, Hosmer Lemeshow χ^2^ statistic was used to assess the calibration of the model with or without sST2. C statistic, net reclassification index (NRI) and integrated discrimination improvement (IDI) were applied to evaluate the predictive ability of plasma sST2 beyond conventional risk factors model, which including age, sex, education level, systolic BP, baseline NIHSS, baseline mRS, hypertension, diabetes mellitus, ischemic stroke subtype, hsCRP, anticoagulant treatment and hypoglycemic treatment. In addition, random forest regression model, one of the most robust ensemble machine learning methods for classification and regression, was used to assess the importance of potential predictors. “Mean Decrease Accuracy” is a type of importance measure and the value is larger suggests that the predictor is more important. Multiple imputation for missing data was performed using the Markov chain Monten Carlo method (Supplementary Table 1). All two-sided *P* values < 0.05 were considered to be statistically significant. Statistical analysis was conducted using SAS statistical software, version 9.4 (SAS Institute Inc., Cary, NC).

## Results

### Baseline characteristics

A total of 619 patients (434 men and 185 women) with a mean (SD) age of 60.0 (10.5) years were enrolled in the present study. The median of plasma sST2 level was 163.51 pg/mL (interquartile range, 117.60–238.77 pg/mL). The baseline characteristics across the quartiles of sST2 levels were summarized in Table 1. Compared to the patients in the lowest quartile of sST2 levels, those in the higher quartile were tended to be male and older, to have higher baseline NIHSS and mRS score, to have higher level of hsCRP, and to have shorter time from onset to randomization and lower proportion of antihypertensive medications use.

**Table 1:** Baseline Characteristics of Participants According to Plasma soluble ST2 (sST2) quartiles

Characteristics^a^	sST2, pg/mL				*p* trend
	< 117.60	117.60–163.51	163.51–238.77	≥238.77	
No. of subjects	155 (25.0)	154 (24.9)	156 (25.2)	154 (24.9)	
**Demographic features**					
Age, y	58.5 ± 10.3	59.9 ± 9.6	59.2 ± 10.3	62.5 ± 11.2	0.003
Male sex, n (%)	88 (56.8)	102 (66.2)	121 (77.6)	123 (79.9)	< 0.001
Education, n (%)					
Illiteracy	8 (5.2)	15 (9.7)	10 (6.4)	18 (11.7)	0.10
Primary	61 (39.4)	64 (41.6)	53 (34.0)	56 (36.4)	0.34
High school	75 (48.4)	68 (44.2)	80 (51.3)	72 (46.8)	0.90
College or higher	11 (7.1)	7 (4.6)	13 (8.3)	8 (5.2)	0.83
Current cigarette smoking, n (%)	55 (35.5)	56 (36.4)	61 (36.5)	53 (34.4)	0.67
Current alcohol drinking, n (%)	51 (32.9)	52 (33.8)	57 (36.4)	53 (34.9)	0.63
**Clinical features**					
Time from onset to randomization, h	12.0 (5.0–24.0)	12.0 (5.0–24.0)	12.0 (6.0–24.0)	6.6 (4.0–24.0)	0.02
Baseline systolic BP, mm Hg	168.3 ± 17.5	166.0 ± 15.2	166.6 ± 16.6	169.0 ± 17.2	0.64
Baseline diastolic BP, mm Hg	98.5 ± 9.6	97.8 ± 9.7	98.2 ± 9.4	98.6 ± 11.4	0.85
Baseline NIHSS score	4.0 (3.0–7.0)	4.0 (2.0–6.0)	4.0 (2.0–7.0)	6.0 (3.0–9.0)	< 0.001
Baseline modified Rankin Scale score	3.0 (2.0–3.0)	3.0 (2.0–4.0)	3.0 (1.0–3.0)	3.0 (2.0–4.0)	< 0.001
High-sensitive C-reactive protein, mg/L	1.3 (0.6–3.7)	1.8 (0.7–4.9)	2.1 (0.9–4.5)	3.8 (1.4–10.0)	< 0.001
**Medical history, n (%)**					
Hypertension	122 (78.7)	120 (77.9)	123 (78.9)	111 (72.1)	0.21
Hyperlipidemia	14 (9.0)	10 (6.5)	8 (5.1)	10 (6.5)	0.32
Diabetes mellitus	30 (19.4)	23 (15.0)	30 (19.2)	21 (13.6)	0.34
Coronary heart disease	12 (7.7)	20 (13.0)	16 (10.3)	18 (11.7)	0.41
Family history of stroke	33 (21.3)	22 (14.3)	22 (14.1)	24 (15.6)	0.19
Use of antihypertensive drugs	75 (48.4)	70 (45.5)	67 (43.0)	58 (37.7)	0.05
Ischemic stroke subtype, n (%)					
Thrombotic	101 (65.2)	101 (65.6)	105 (67.3)	95 (61.7)	0.61
Embolic	4 (2.6)	5 (3.3)	5 (3.2)	9 (5.8)	0.15
Lacunar	50 (32.3)	48 (31.2)	46 (29.5)	50 (32.5)	0.95
Receiving immediate BP reduction	67 (43.2)	81 (47.4)	83 (53.2)	72 (46.8)	0.53
Anticoagulant treatment	35 (22.6)	32 (20.8)	36 (23.1)	48 (31.2)	0.07
Hypoglycemic treatment	23 (14.8)	28 (18.2)	32 (20.5)	22 (14.3)	0.96

*Abbreviations*: *BP* blood pressure, *NIHSS* National Institute of Health Stroke Scale

^a^Continuous variables are expressed as mean ± standard deviation or median (interquartile range). Categorical variables are expressed as frequency (%)

### Association between plasma sST2 and PSCI

**Fig. 1:**
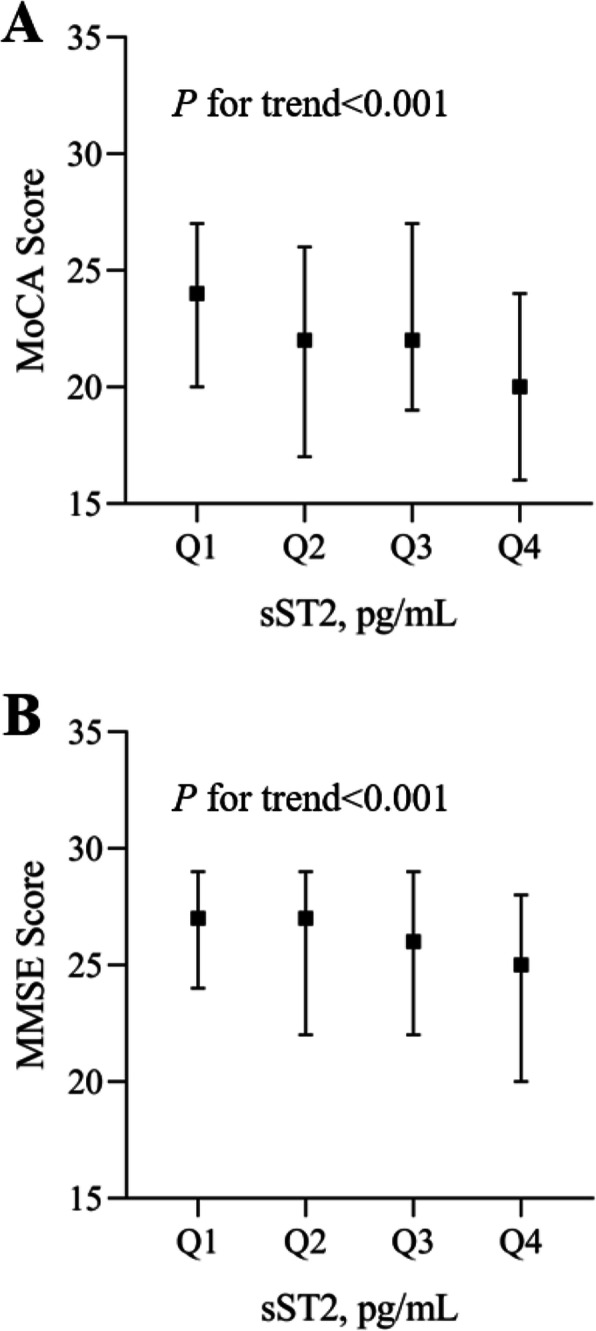
MoCA and MMSE score in acute ischemic stroke patients by sST2 quartiles. Panel **a** MoCA score; Panel **b** MMSE score. MoCA: Montreal Cognitive Assessment; MMSE: Mini-Mental State Examination; sST2: soluble suppression of tumorigenicity 2

At 3-month follow-up, the median (interquartile range) score of MoCA and MMSE were 22.0 (18.0–26.0) and 26.0 (22.0–29.0), respectively. There were significantly decreasing trends in MoCA and MMSE scores as sST2 increased from the lowest quartile to the highest quartile (Fig. 1). 325 (52.5%) or 323 (52.2%) participants developed PSCI according to MoCA or MMSE, respectively. The incidences of PSCI increased from the lowest quartile (sST2 < 117.60 pg/mL) to the highest quartile (sST2 ≥ 238.77 pg/mL) (Table 2).

**Table 2:** ORs and 95% CIs for the Risk of post-stroke cognitive impairment According to sST2 quartiles

	sST2, pg/mL				*p* value for trend
	< 117.60	117.60–163.51	163.51–238.77	≥238.77	
Median	89.75	139.13	196.13	349.14	
**Cognitive impairment: MoCA**					
Cases, n (%)	63 (40.7)	79 (51.3)	81 (51.9)	102 (66.2)	< 0.001
Model 1	1.00	1.48 (0.93–2.35)	1.56 (0.98–2.48)	2.53 (1.55–4.12)	< 0.001
Model 2	1.00	1.57 (0.96–2.55)	1.64 (1.01–2.68)	2.35 (1.40–3.93)	0.002
Model 3	1.00	1.58 (0.97–2.56)	1.65 (1.01–2.70)	2.38 (1.42–4.00)	0.002
**Cognitive impairment: MMSE**					
Cases, n (%)	69 (44.5)	73 (47.4)	81 (51.9)	100 (64.9)	< 0.001
Model 1	1.00	1.04 (0.66–1.66)	1.28 (0.80–2.04)	1.93 (1.19–3.14)	0.003
Model 2	1.00	1.09 (0.67–1.76)	1.31 (0.81–2.12)	1.76 (1.06–2.92)	0.021
Model 3	1.00	1.10 (0.68–1.78)	1.33 (0.82–2.16)	1.82 (1.09–3.03)	0.016

MoCA score of < 23 or MMSE score of < 27 indicates cognitive impairment

Model 1: adjusted for age, sex, and education level;

Model 2: adjusted for model 1 and further adjusted for current smoking, alcohol drinking, time from onset to randomization, systolic blood pressure, baseline National Institutes of Health Stroke Scale score, baseline modified Rankin Scale score, medical history (hypertension, diabetes mellitus, hyperlipidemia, and coronary heart disease), use of antihypertensive medications, randomized treatment, ischemic stroke subtype, anticoagulant treatment and hypoglycemic treatment

Model 3: adjusted for model 2 and further adjusted for hsCRP

*Abbreviations*: *sST2* soluble suppression of tumorigenicity 2, *MoCA* Montreal Cognitive Assessment, *MMSE* Mini-Mental State Examination, *OR* odds ratio

After adjustment for age, sex, education level, current smoking, alcohol drinking, time from onset to randomization, systolic BP, baseline NIHSS and mRS score, medical history (hypertension, diabetes mellitus, hyperlipidemia, and coronary heart disease), use of antihypertensive medications, randomized treatment, ischemic stroke subtype, anticoagulant treatment and hypoglycemic treatment (model 2), the odds of developing PSCI (defined by MoCA score) increased significantly with elevated baseline sST2 levels (*p* for trend = 0.002). This significant association remained when further adjustment for hsCRP levels (model 3). Compared to patients in the lowest quartile of sST2, those in the highest quartile had 2.38-fold (95% CI 1.42–4.00) risk of PSCI. Similar independent association was also observed when PSCI was defined according to MMSE score, the corresponding adjusted OR (95% CI) of PSCI was 1.82 (1.09–3.03) for patients in the highest quartile of sST2 in comparison with those in the lowest quartile of sST2 (Table 2).

### Incremental predictive value of plasma sST2

The Hosmer Lemeshow test showed that model calibration was adequate after adding plasma sST2 to the basic model containing conventional risk factors (MoCA *p* = 0.20; MMSE *p* = 0.74). Incorporation sST2 into the basic model also significantly improved the risk reclassification performance (continuous NRI 18.5%; IDI 1.6%; both *p* < 0.01) when PSCI was defined by MoCA score (Table 3). Similar incremental predictive value of sST2 was observed when PSCI was evaluated by MMSE score.

**Table 3:** Reclassification Statistics (95% CI) for post-stroke cognitive impairment by plasma sST2 Among Participants

	C statistic		Calibration statistic		NRI (Continuous)		IDI	
	Estimate (95% CI)	*p* value	χ^2^	*p* value	Estimate (95% CI), %	*p* value	Estimate (95% CI), %	*p* value
**Cognitive impairment: MoCA**								
Conventional model	0.678 (0.640 to 0.715)		6.12	0.63	Reference		Reference	
Conventional model + sST2 (quartiles)	0.691 (0.653 to 0.727)	0.07	11.10	0.20	18.5 (3.0 to 33.9)	< 0.01	1.6 (0.6 to 2.6)	< 0.01
**Cognitive impairment: MMSE**								
Conventional model	0.672 (0.633 to 0.708)		1.42	0.99	Reference		Reference	
Conventional model + sST2 (quartiles)	0.683 (0.644 to 0.719)	0.11	5.19	0.74	17.8 (2.1 to 33.4)	< 0.01	1.0 (0.2 to 1.8)	< 0.01

MoCA score of < 23 or MMSE score of < 27 indicates cognitive impairment

Conventional model included age, sex, education level, systolic blood pressure, baseline National Institutes of Health Stroke Scale score, baseline modified Rankin Scale score, hypertension, diabetes mellitus, ischemic stroke subtype, hsCRP, anticoagulant treatment and hypoglycemic treatment

*Abbreviations*: *sST2* soluble suppression of tumorigenicity 2, *CI* confidence interval, *IDI* integrated discrimination index, *MoCA* Montreal Cognitive Assessment, *NRI* net reclassification improvement

**Fig. 2:**
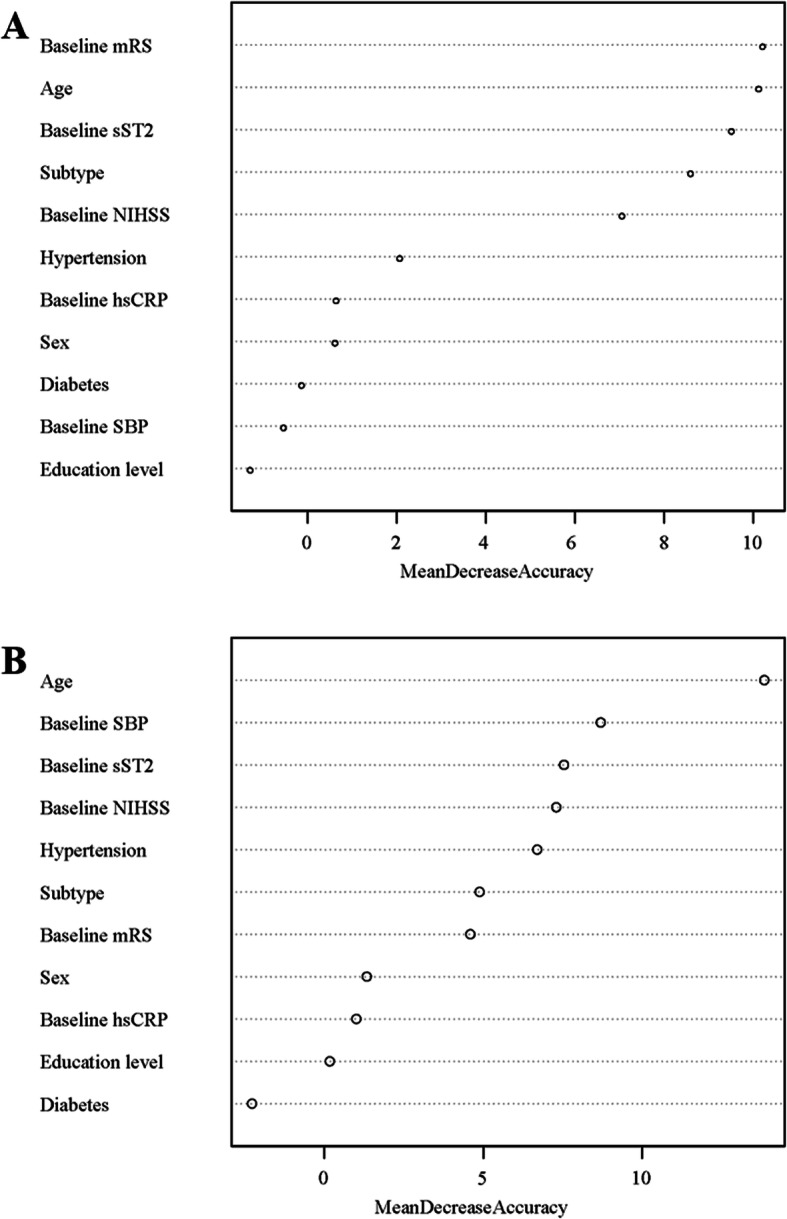
Potential predictors importance analyses for Post-stroke cognitive impairment. Panel **a** Montreal Cognitive Assessment (MoCA) score < 23; Panel **b** Mini-Mental State Examination (MMSE) score < 27. mRS: modified Rankin Scale score; sST2: soluble suppression of tumorigenicity 2; NIHSS: National Institutes of Health Stroke Scale score; hsCRP: high-sensitive C-reactive protein; SBP: systolic blood pressure. Mean Decrease Accuracy: a type of importance measure and the value is larger suggests that the predictor is more important

Additionally, to test the contribution of potential predictors in relation to PSCI, mean decrease in accuracy was calculated. Moreover, we found that sST2 was superior to NT-proBNP in predicting PSCI, according to the receiver operating characteristic curves (Supplementary Figure 2). The random forest regression models suggested that baseline plasma sST2 was one of the promising predictors for PSCI (Fig. 2).

## Discussion

This prospective study using data from CATIS trial found that higher plasma sST2 levels were associated with increased risk of PSCI, independently of potential confounders including education, stroke severity and medical history. The significant association remained when further controlled inflammatory biomarker. In addition, adding sST2 levels into the model containing conventional risk factors statistically improved the predictive ability, as evidenced by NRI and IDI statistic. Furthermore, sST2 was one of the promising predictors for PSCI. These findings provided population-based evidence of plasma sST2 as a potential biomarker in predicting PSCI.

Emerging evidence from epidemiological studies support that sST2 is of diagnostic and prognostic value in the setting with various cardiovascular diseases, including heart failure, coronary artery disease, and ischemic stroke [14, 23–26]. For example, the Linz Stroke Unit Study conducted in acute ischemic stroke patients reported a higher level of sST2 in decedents than survivors [24]. Furthermore, Wolcott et al. demonstrated that sST2 was an independent predictor of short-term mortality, functional outcome and hemorrhagic transformation in patients with ischemic stroke [25]. Of note, patients with cardiovascular diseases are at higher risk of experiencing cognitive decline. However, clinical studies designed to specifically investigate the association between sST2 and cognitive impairment, especially in the condition of ischemic stroke, are sparse.

A small study of 18 mild cognitive impairment patients and 17 healthy controls showed that serum sST2 levels were significantly higher in patients with mild cognitive impairment than controls [27]. Furthermore, Andersson et al. using the data from the Framingham Offspring Study reported a cross-sectional association between sST2 concentrations and cognitive impairment, and they found participants in the highest quartile of sST2 had significantly lower brain volumes and poorer delayed performance on the visual reproduction test than those in the lowest quartile [14]. Similarly, previous studies also suggested significant associations between other cardiac biomarkers with neurological disorders. For example, a cross-sectional analysis of 860 ischemic stroke patients suggested that hs-cTnT was associated with the severity of white matter lesions, which was considered as a predictor of poorer cognitive function [28, 29]. Recently, the PROSCIS-B (Prospective Cohort With Incident Stroke Berlin) study reported that higher hs-cTnT was associated with higher prevalence of cognitive impairment at baseline and lower Telephone Interview for Cognitive Status-modified during 3-year follow-up in patients with mild-to-moderate ischemic stroke [20]. The present study, to our knowledge, was the first longitudinal study to directly characterize the relationship of sST2 and PSCI, extending the connection of heart and brain to the patients with ischemic stroke.

The mechanisms underlying the sST2-PSCI association are still unclear, but several potential pathophysiological pathways have been proposed. Cardiac dysfunction was implicated in various pathological conditions, including hemodynamic stress, cerebral hypoperfusion, neuroinflammation, cardiac arrhythmias, and hypercoagulation, and then may further lead to cognitive impairment [6, 7]. Moreover, prior studies showed that cerebral small vessel disease (CSVD) and brain atrophy had relationship with cognitive dysfunction [30, 31]. As a serum cardiac marker, sST2 could indicate the load of the CVSD [7] and elevated sST2 was associated with lower brain volumes [14], which might affect cognitive function. Furthermore, interleukin 33 was found to be neuroprotective in experimental stroke models [32], and the administration of interleukin 33 could reduce cognitive decline [27]. Inflammation might be the potential mechanisms. CRP, a typical inflammatory marker, can induce other proinflammatory factors and was associated with an increased risk of stroke [33, 34]. However, after additionally adjusting hsCRP in the Model 3, the significant relationships remained. Further studies are required to clarify related mechanism.

Several lines of evidence suggested that age, sex, education attainment, admission BP, stroke severity, medical history, ischemic stroke subtype, and inflammation were associated with cognitive status in the general population or participants with cardiovascular disease [35–39]. In the present study, the sST2-PSCI relationship remained after adjustment for these established risk factors, indicating sST2 independently contributed to the risk of PSCI. This might relate to plasma sST2 reflecting heart and brain injury, and its specificity for cardiac function might distinguish plasma sST2 from other blood-based biomarkers with predictive value for PSCI reflecting other biological processes, such as tHcy, RF, MMP-9 and TIMP-1 [40–42].

In addition, incorporating sST2 into a model with known risk factors statistically improved reclassification for PSCI prediction. Moreover, sST2 was one of the promising predictors for PSCI. Therefore, the evaluation of the association between sST2 and PSCI had important clinical significance given the high prevalence and heavy disease burden of PSCI. These findings, coupled with the evidence that sST2 was of prognosis value in ischemic stroke patients, imply the clinical usefulness of sST2 measurement to identify patients at high risk of PSCI and provide novel therapeutic interventions. Future well-designed clinical trials aimed to test the effect of inhibition of sST2 treatment on cognitive impairment among ischemic stroke patients are warranted.

Our study was based on a subsample of the well-performed CATIS trial with standardized protocol and rigid quality control procedures, enabling us to provide a more comprehensive and valid assessment of the association between plasma sST2 levels with PSCI. However, our study has several limitations. First, patients with BP ≥220/120 mmHg or treatment with intravenous thrombolytic therapy at admission were not included in the CATIS trial. These limited the generalizability of our findings to all acute ischemic strokes. Second, plasma sST2 levels were only measured once at admission, we could not explore its dynamic changes over time and the effect on PSCI. Third, we did not collect the information of pre-stroke cognitive status due to lack of feasibility. However, we included NIHSS score at admission in the multivariate model, which had a subset cognitive dysfunction evaluation and had almost the same diagnostic value as the MMSE (area under the ROC curve values of 0.78 and 0.84, respectively) [43]. Finally, the data of brain and cardiac imaging, such as the site or type of acute ischemic lesions, left atrial volume or left ventricular dysfunction were also not recorded. Hence, we could not further control these factors.

## Conclusions

In conclusion, elevated concentrations of plasma sST2 levels were significantly associated with cognitive impairment in acute ischemic stroke patients, independently of established risk factors. Our findings provided additional information for early identifying patients at increased risk of PSCI.

## Supplementary Information

**Additional file 1: Supplementary Figure 1.** Study participant flow chart. **Supplementary Figure 2.** Comparison of ROC curves for sST2 vs NT-proBNP in predicting PSCI. **Supplementary Table 1.** Missing variable in the study. **Supplementary Table 2.** Baseline characteristics of acute ischemic stroke patients.

## Data Availability

The datasets used and/or analyzed during the current study are available from the corresponding author on reasonable request.

## Abbreviations

sST2: Soluble suppression of tumorigenesis-2
MoCA: Montreal Cognitive Assessment
MMSE: Mini-Mental State Examination
PSCI: Post-stroke cognitive impairment
NT-proBNP: N-terminal pro-B-type natriuretic peptide
hs-cTnT: High-sensitivity cardiac Troponin T
CATIS: China Antihypertensive Trial in Acute Ischemic Stroke
BP: Blood pressure
hsCRP: High-sensitive C-reactive protein
NIHSS: National Institutes of Health Stroke Scale
mRS: Modified Rankin Scale
ORs: Odds ratios
CIs: Confidence intervals
NRI: Net reclassification index
IDI: Integrated discrimination improvement
SD: Standard deviation
CSVD: Cerebral small vessel disease
