# Supplementary material for: Soluble ST2 and risk of cognitive impairment after acute ischemic stroke: a prospective observational study
Source: BMC Geriatr. 2021 May 24;21:330. doi: 10.1186/s12877-021-02288-6 (PMC8142507; doi:10.1186/s12877-021-02288-6)
Supplement: Supplementary file 1 — Additional file 1: Supplementary Figure 1. Study participant flow chart. Supplementary Figure 2. Comparison of ROC curves for sST2 vs NT-proBNP in predicting PSCI. Supplementary Table 1. Missing variable in the study. Supplementary Table 2. Baseline characteristics of acute ischemic stroke patients. [file 12877_2021_2288_MOESM1_ESM.docx]

**Title Page**

**Title: Soluble ST2 and risk of cognitive impairment after acute ischemic stroke:** **a prospective observational study**

Yinwei Zhu, MD^1, *^; Chongquan Fang, MD^1, *^; Qi Zhang, MD^1, *^; Yaling Lu, MD^1^;

Rui Zhang, MD^1^; Aili Wang, MD, PhD^1^; Xiaoqing Bu, MD, PhD^2^; Jintao Zhang, MD^3^; Zhong Ju, MD, PhD^4^; Yonghong Zhang, MD, PhD^1^; Tan Xu, MD, PhD^1^**^#^**;

Chongke Zhong, MD, PhD^1^**^#^**

^1^Department of Epidemiology, School of Public Health and Jiangsu Key Laboratory of Preventive and Translational Medicine for Geriatric Diseases, Medical College of Soochow University, Suzhou, China

^2^Department of Epidemiology, School of Public Health, Chongqing Medical University, Chongqing, China

^3^Department of Neurology, The 88th Hospital of PLA, Shandong, China

^4^Department of Neurology, Kerqin District First People’s Hospital of Tongliao City, Tongliao, China

^*^**These authors contributed equally to this work.**

**^#^ Correspondence authors:**

Chongke Zhong, MD, PhD or Tan Xu, MD, PhD

Department of Epidemiology, School of Public Health and Jiangsu Key Laboratory of Preventive and Translational Medicine for Geriatric Diseases, Medical College of Soochow University, 199 Renai Road, Industrial Park District, Suzhou, Jiangsu Province 215123, China. E-mail: ckzhong@suda.edu.cn; [xutan@suda.edu.cn](mailto:xutan@suda.edu.cn).

**Cover title:** Soluble ST2 and Post-stroke cognitive impairment

**Additional file：**Supplementary Figures: 2; Supplementary Tables: 2.

**Supplementary material**

**Supplementary Figure 1** Study participant flow chart.


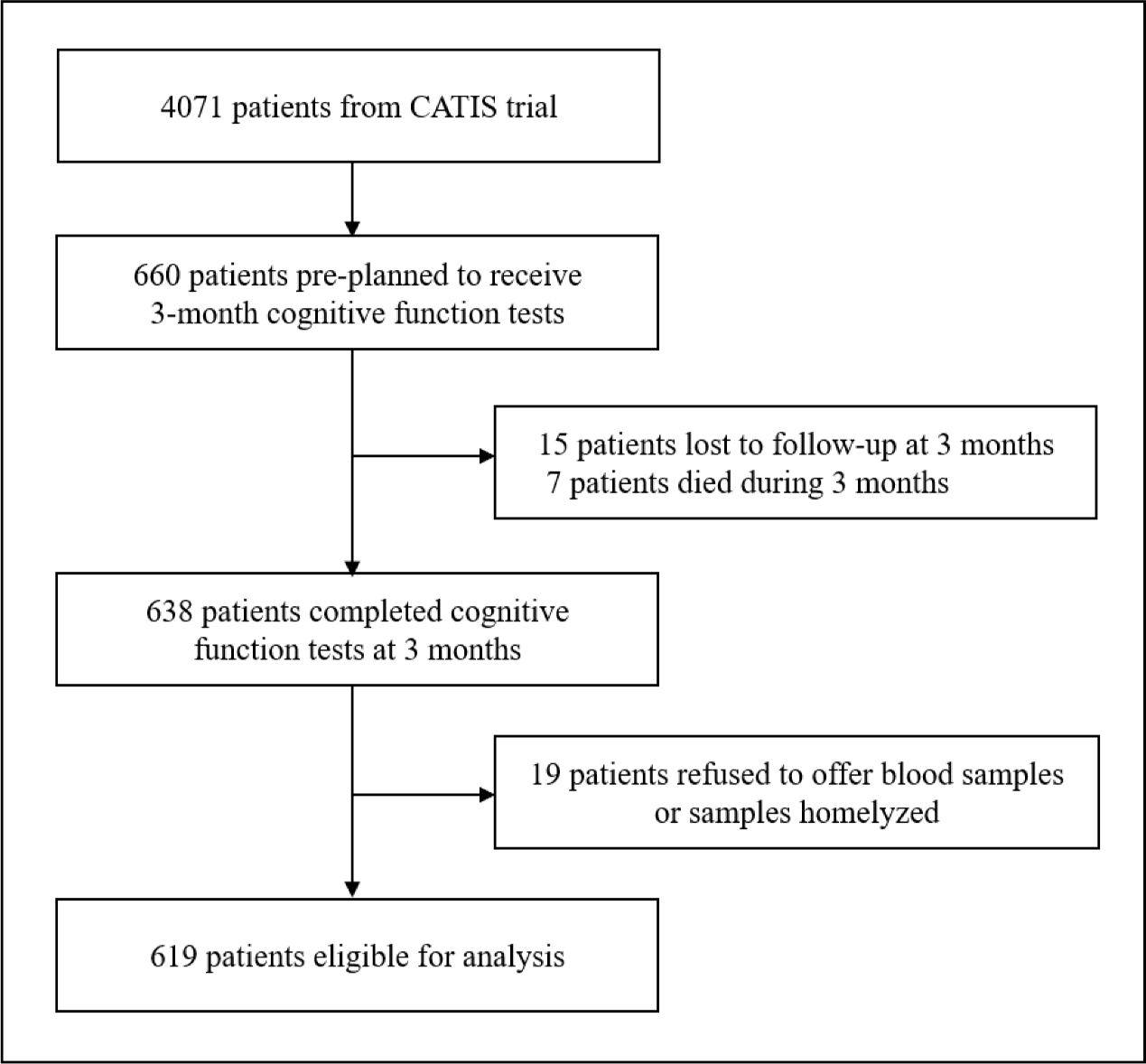
CATIS: China Antihypertensive Trial in Acute Ischemic Stroke

**Supplementary Figure 2** Comparison of ROC curves for sST2 vs NT-proBNP in predicting PSCI.


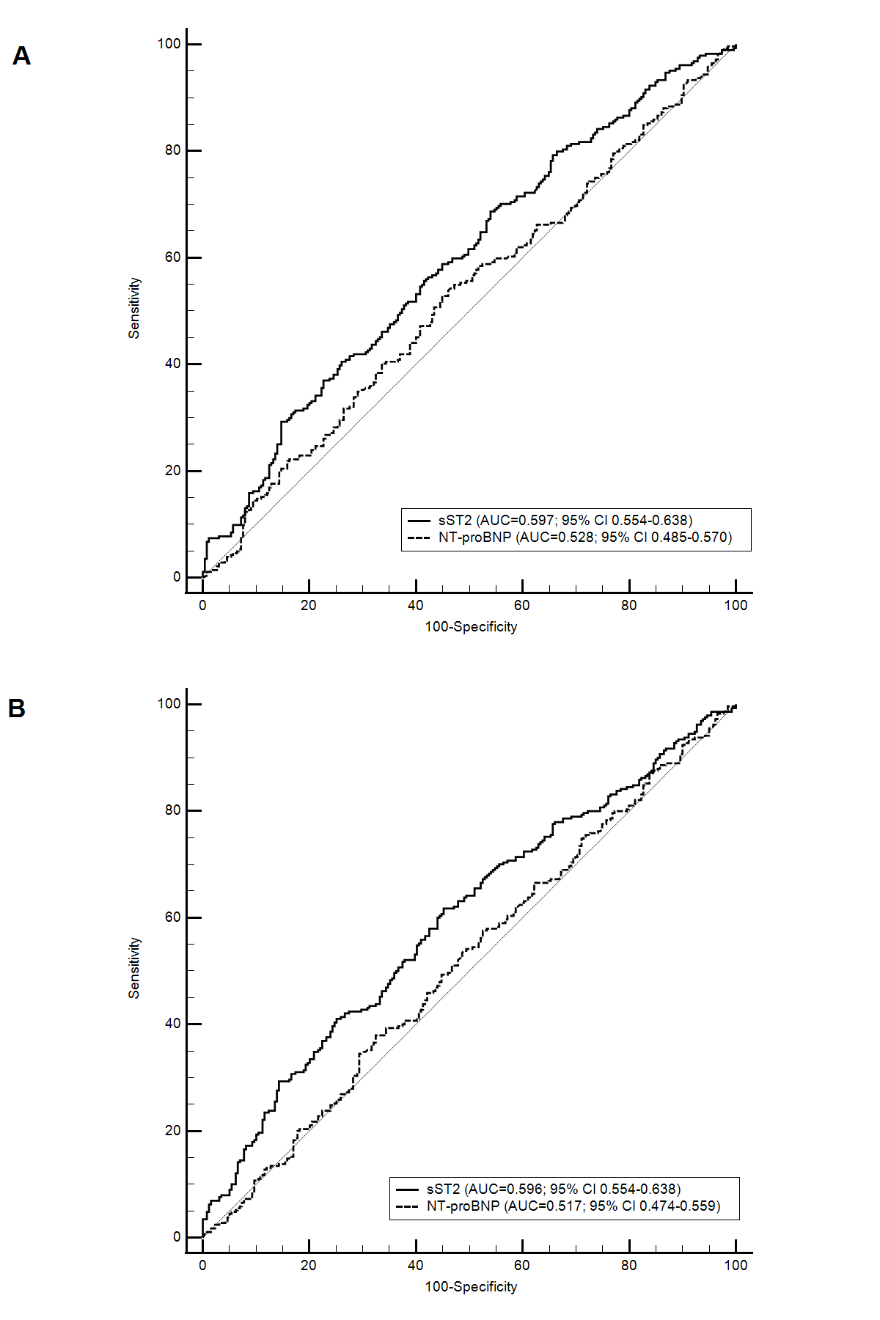


Panel A: MoCA score; Panel B: MMSE score.

ROC: Receiver operating characteristic; PSCI: Post-stroke cognitive impairment;

MoCA: Montreal Cognitive Assessment; MMSE: Mini-Mental State Examination; sST2: soluble suppression of tumorigenicity 2; NT-proBNP: N-terminal pro-B-type natriuretic peptide; CI: Confidence interval; AUC: Area under the ROC curve.

**Supplementary Table 1** Missing variable in the study.

| Variable | Total | Missing(%) | Variable | Total | Missing(%) |
| --- | --- | --- | --- | --- | --- |
| Age | 619 | 0 (0.00) | Education level | 619 | 0 (0.00) |
| Baseline diastolic BP | 619 | 0 (0.00) | Family history of stroke | 619 | 0 (0.00) |
| Baseline hsCRP | 579 | 40 (6.46) | Hyperlipidemia | 619 | 0 (0.00) |
| Baseline mRS | 619 | 0 (0.00) | Hypertension | 619 | 0 (0.00) |
| Baseline NIHSS | 619 | 0 (0.00) | Receiving immediate BP reduction | 619 | 0 (0.00) |
| Baseline systolic BP | 619 | 0 (0.00) | Sex | 619 | 0 (0.00) |
| Baseline sST2 | 619 | 0 (0.00) | Smoking | 619 | 0 (0.00) |
| Coronary heart disease | 619 | 0 (0.00) | Stroke subtype | 619 | 0 (0.00) |
| Diabetes mellitus | 619 | 0 (0.00) | Time from onset to randomization | 616 | 3 (0.48) |
| Drinking | 619 | 0 (0.00) | Use of antihypertensive drugs | 619 | 0 (0.00) |

BP: blood pressure; hsCRP: high-sensitive C-reactive protein; mRS: modified Rankin Scale score; NIHSS: National Institutes of Health Stroke Scale score; sST2: soluble suppression of tumorigenicity 2.

**Supplementary Table 2** Baseline characteristics of acute ischemic stroke patients

| Characteristics | Excluded | Enrolled | p value |
| --- | --- | --- | --- |
| Number of patients | 3452 | 619 |  |
| **Demographics** |  |  |  |
| Age, y | 62.3 ± 10.9 | 60.0 ± 10.5 | <0.001 |
| Male sex | 2170 (62.9) | 434 (70.1) | <0.001 |
| Education level |  |  | <0.001 |
| Illiteracy | 491 (14.2) | 51 (8.2) |  |
| Primary | 1306 (37.8) | 234 (37.8) |  |
| High school | 1482 (42.9) | 295 (47.7) |  |
| College or higher | 173 (5.0) | 39 (6.3) |  |
| Current alcohol drinking | 1040 (30.1) | 213 (34.4) | 0.034 |
| Current cigarette smoking | 1252 (36.3) | 233 (37.6) | 0.51 |
| **Clinical features** |  |  |  |
| Time from onset to randomization, h | 10.0 (4.5-24.0) | 10.0 (5.0-24.0) | 0.85 |
| Baseline systolic BP, mm Hg | 165.9 ± 16.9 | 167.5 ± 16.7 | 0.032 |
| Baseline diastolic BP, mm Hg | 96.4 ± 11.3 | 98.3 ± 10.0 | <0.001 |
| Body mass index, kg/m^2^ | 24.8 (22.9-26.8) | 24.7 (22.9-26.5) | 0.66 |
| Baseline NIHSS score | 4.0 (2.0-8.0) | 4.0 (3.0-7.0) | 0.16 |
| Baseline modified Rankin Scale score | 3.0 (2.0-4.0) | 3.0 (2.0-4.0) | 0.16 |
| High-sensitive C-reactive protein, mg/L | 2.5 (0.8-8.3) | 2.1 (0.8-5.2) | 0.001 |
| **Medical history** |  |  |  |
| Hypertension | 2733 (79.2) | 476 (76.9) | 0.20 |
| Coronary heart disease | 378 (11.0) | 66 (10.7) | 0.83 |
| Diabetes mellitus | 615 (17.8) | 104 (16.8) | 0.54 |
| Hyperlipidemia | 235 (6.8) | 42 (6.8) | 0.98 |
| Family history of stroke | 652 (18.9) | 101 (16.3) | 0.13 |
| Use of antihypertensive drugs | 1727 (50.0) | 270 (43.6) | 0.003 |
| SUBTYPE |  |  | <0.001 |
| Thrombotic | 2742 (79.4) | 402 (64.9) |  |
| Embolic | 171 (5.0) | 23 (3.7) |  |
| Lacunar | 539 (15.6) | 194 (31.3) |  |
| Receiving immediate BP reduction | 1735 (50.3) | 303 (49.0) | 0.55 |
| Anticoagulant treatment | 1222(35.4) | 151(24.4) | <0.001 |
| Hypoglycemic treatment | 608(17.6) | 105(17.0) | 0.70 |

Abbreviations: BP, blood pressure; NIHSS, National Institute of Health Stroke Scale.

*Continuous variables are expressed as mean ± standard deviation or median (interquartile range). Categorical variables are expressed as frequency (%).
